# Parental Psychological Abuse toward children and Mental Health Problems in adolescence

**Published:** 2014

**Authors:** Syeda Fariha Iram Rizvi, Najma Najam

**Affiliations:** 1 Syeda Fariha Iram Rizvi, Institute of Psychology, University of Punjab, Lahore, Pakistan.; 2 Dr. Najma Najam, Institute of Psychology, University of Punjab, Lahore, Pakistan.

**Keywords:** Adolescents, Mental health problems, Psychological abuse

## Abstract

***Objective: ***Present study was conducted to explore the relationship between parental psychological abuse toward their children and mental health problems in adolescence.

***Method: ***Three hundred participants age range 13-17 years, (57% boys and 43% girls) participated in the study from both public and private high schools of Lahore. Psychological maltreatment experience scale (PMES) and Youth Self-Report(YSR) were used for assessment and diagnosis.

***Results:*** Findings revealed that psychological abuse by parents significantly related with mental health problems in adolescents, for mother abuse (r= .24 to.67, p< .05) and father abuse (r= .20 to.70, p< .05). Adolescents who perceived their parents as more abusive exhibited greater problems. Regression analysis indicated that hypothesized factors of parental psychological abuse predicted the mental health problems in adolescents (contributed from 10% to 49% of variance).

***Conclusion: ***Psychological abuse by parents is related with mental health problems in adolescents. These findings will contribute to a better understanding of non-injurious psychological abuse and its impact on adolescents. Findings of the study can be used to bring the attention of parents, public and professionals’ towards damaging effects of psychological abuse on adolescents

## INTRODUCTION

Psychological abuse (PA) is under recognized and under reported phenomena especially in the adolescents by their own parents. It has been described as the most challenging as well as the most prevalent form of child abuse.^1^ PA is rather difficult to define and assess as compare to physical abuse^2^ and may be described as, verbal abuse, harsh nonphysical punishments, or threats of abuse.^3^ It describes a repeated pattern of adult-to-child behavior (usually a parent) that makes the child feel worthless, flawed, unloved, unwanted, endangered, or only of value in meeting another’s needs.^4^

In many cases PA is considered to be the most developmentally damaging dimension^5^ and has been linked with negative outcomes such as impaired emotional, social, and cognitive development, including helplessness, aggression, emotional unresponsiveness and neuroticism.^6^ Research consistently suggests that PA and neglect in childhood have negative effects on normal development.^7^ The experiences of abuse and neglect not only put adolescents at risk for immediate adverse consequences such as poor school performance and increased psychological distress^8^ but may also have long-term serious health outcomes such as delinquency, aggression, low self-esteem, anxiety, substance abuse**, **post traumatic stress disorder (PTSD), and depression.^9^ PA is also explained in terms of abuse of adolescents’ mental and emotional process which has been linked with psychiatric disorders in childhood such as major depression and dysthymia.^10^Of the few studies documenting its long-term effects, Mullen and colleagues^11^ report that childhood PA relates to eating disorders, depressive illness, and suicidal behaviors in adulthood.

Recent researches has also shown that children who have experienced PA exhibit common mental health problem including depression, anxiety, low-self-esteem, and eating disorders.^4^ In turn, these problems are associated with physical health problems, including overall poor physical health, increased risk of heart disease, self-injurious behaviors.^3^ This type of abuse can be extremely destructive and has been associated with a range of adverse child outcomes including emotional maladjustment, depression, poor self-esteem, conduct problems, aggression, inability to trust, and underachievement.^12^

In Pakistani context child abuse still remains a taboo subject and most of the cases at home go unreported therefore, help from a mental health expert neither recognized nor accepted rather still is considered to be a social stigma.^13^ In last few years, some attempts have been made to study different dimensions of mental health problems among school children in Pakistan.^14^ Hussein^15^ indicated that 42.30% were rated having conduct problems based on parental report. In another study parents reported 34.40% on prevalence of emotional and behavioral problems of children, whereas teacher reported 35.80% on “Abnormal category”.^16^ keeping in view the fact that there are life-long consequences of child abuse, the present investigation aimed to identify mental health problems of abused adolescents. 

***Significance of the study:*** Child’s protection rights include protection from psychological abuse because PA is considered less serious and significant as compared to physical and sexual abuse.^17^ With observations that its prevalence is increasing, and a growing understanding of its profound serious effects on healthy development, PA has become an issue of concern globally.^1^ The results of the present research will address the existing unknown, and suspected cases of psychological abuse by parents and behavioral problems. 

***Hypotheses:***

1. There will be significant positive relationship between perceived psychological abuse by parents (mother & father) and mental health problems in adolescents.

2. Factors of Psychological abuse are likely to be predictors of mental health problems.

## METHOD

***Research Design and Participants: ***Co relational research design (within subject research design) was used to find the relationship between perception of parental psychological abuse and mental health problems in adolescents. Participants (N=300) were selected from both private and public schools (secondary) of three towns of Lahore through stratified random sampling. Secondary schools were further divided into three categories, only boy’s schools, only girl’s schools, and co-educational schools. Further classification was based on age range (13-17years) and class (7^th^ -10^th^). Participants were chosen through purposive sampling. Teachers were requested to select those students who exhibited some problem behavior as well as appeared neglected from parents. 

***Measures***

***Demographic Information Form: ***Students completed a questionnaire inquiring about gender, age, parental education, school system etc.

***Psychological maltreatment experience scale*** ^18^ (PMES), consisted on 53 items (α .97) was translated into Urdu, Mother Form (α= .96) and Father Form(α= .95), asks respondents to rate the frequency with which they were subjected to five types of psychological abuse (i.e., verbal abuse/attacks on self-worth; neglectful/rejecting behaviors; withholding supportive behaviors; minimizing, isolating, and terrorizing; and, exploitative parental behaviors) byparents. Each item was rated on four point Likert scale (never=*1*, occasionally=*2*, fairly often=*3*, or very often=*4*).

***Youth Self-Report (YS*** ^19^ ***): ***Urdu translation of YSR, consisted on 112 items is a 3-point scale (*0=not true, 1=somewhat true, 2= very true or often true*), was used to identify mental health problems. It measures six mental health /DSM oriented problems (Diagnostic and Statistics Manual for Mental Disorders; DSM-IV^20^),consisted of affective problems, anxiety problems, somatic problems, attention deficit hyperactivity problems, oppositional defiant problems, and conduct problems.

***Procedure:*** Due Permission was taken from authors of questionnaires to translate into Urdu and to use. Permission was also taken the from Education Department and schools authorities to collect the data from schools. Class teachers of required age group (adolescents) were approached and briefed about study purpose and with the help of teachers adolescents in the required age group were selected. Informed consent was taken from those adolescents whose parents allow them to participate in the study*. *After detailed verbal instruction participants were given the questionnaires. Researcher was available to clarify and to answer the questions that students might have about the study and the questionnaire. The questionnaires were self-administered and took about 20 minurtes to complete.

## RESULTS

In order to test the hypotheses, data were screened for basic assumptions (e.g. adequate sample size, Singularity, normality, linearity & homoscedasticity) of correlation and regression prior to analysis. Pearson correlation was carried out on the major variables, where relationship was predicted. To identify the significant predictors of outcome variables (mental health problems) regression analyses were carried out.

Demographic characteristics of sample (Table-I) shows that only school going adolescent girls (43%) and boys (57%) were selected from intact families, who were never reported for abuse as well as for problem behaviors. Findings of the study indicated that perceptions of PA by mother and father and its all factors (verbal abuse, neglectful/rejecting behavior, withholding support, terrorizing and exploitative behavior) have significant positive relationship with mental health problems (affective problems, anxiety problems, attention deficit/hyperactivity problems, oppositional defiant disorder, conduct problem, obsessive compulsive problem, and post-traumatic stress problem). Adolescents perceived their parents more abusive significantly exhibit greater mental health problems as categorized through DSM IV. Correlation range from r= .24 (exploitative mother with obsessive compulsive problem) to .67 (total psychological abuse with affective problem, oppositional defiant disorder and conduct problem), (p< .05) for mother abuse and r= .20 (exploitative father with obsessive compulsive problem) to.70,(total psychological abuse with conduct problem), (p< .05) for father abuse (Table-II & III). Findings of regression analysis indicated that different factors of psychological abuse of mother and father predict different emotional and behavioral problems. Most of the factors of parental PA appeared as significant predictors of mental health problems but mother’s verbal abuse and father’s terrorizing behavior were most common predictors for many problems. Prediction value (R^2^=.10 to .49) for different problems indicated that factors of parental PA is contributing from 10% to 49% in the different mental health problems (Table- IV). All the models were significant (p< .001). Findings of correlation and regression analysis are reported in the following tables. 

**Table-I:** Demographic characteristics of sample

*Variables*		*F*	*%*	*Variables*		*F*	*%*
Age in years	13-17 M= 14.8	300		Father education	Less than or high school	148	49
Class/grade	7^th^-10^th^				College/ Graduation Masters or above	102 50	34 17
Gender	Boys	170	57		Less than or High School	178	59
	Girls	130	43				
School system	Private	186	62	Mother education	College/Graduation	100	33
	Public/Government	114	38		Masters or above	22	8

***Note:*** F= frequency, %= percentage

**Table-II:** Correlation between Perception of Perceived Psychological Abuse by Mother and Mental Health Problems among Adolescents (N=300).

	*MVA*	*NRM*	*WSM*	*TM*	*EM*	*TPA*
Affective problem	.66**	.65**	.36**	.62**	.55**	.67**
Anxiety problem	.54**	.56**	.28**	.53**	.49**	.56**
Attention deficit hyperactivity problem	.58**	.56**	.28**	.56**	.50**	.58**
Oppositional defiant disorder	.66**	.64**	.37**	.63**	.57**	.67**
Conduct problem	.65**	.66**	.36**	.63**	.57**	.67**
Obsessive compulsive problem	.31**	.25**	.26**	.28**	.24**	.30**
Post traumatic stress problem	.63**	.62**	.36**	.61**	.54**	.64**

MVA= Mother Verbal Abuse, NRM= Neglectful/Rejecting Mother, WSM, Withholding Support Mother, TM= Terrorizing Mother, EM= Exploitative Mother, TPA= Total Psychological Abuse

** P< .01.

**Table-III:** Correlation between Perception of Perceived Psychological Abuse by Father andMental Health Problems among Adolescents (N=300).

	*FVA*	*NRF*	*WSF*	*TF*	*EF*	*TPA*
Affective problem	.64**	.64**	.32**	.32**	.63**	.67**
Anxiety problem	.53**	.54**	.27**	.27**	.50**	.56**
Attention deficit hyperactivity problem	.58**	.57**	.32**	.32**	.57**	.61**
Oppositional defiant disorder	.67**	.64**	.32**	.32**	.65**	.69**
Conduct problem	.66**	.67**	.67**	.31**	.66**	.70**
Obsessive compulsive problem	.26**	.23**	.24**	.24**	.20**	.28**
Post-traumatic stress problem	.62**	.59**	.34**	.60**	.51**	.64**

FVA= Father Verbal Abuse, N/RF= Neglectful/Rejecting Father, WSF, Withholding Support Father, TF= Terrorizing Father, EF= Exploitative Father, TPA= Total psychological abuse

** P< .01.

**Table-IV:** Regression analysis predicting DSM oriented Mental Health problems in adolescents from psychological abuse by both mother and father (verbal abuse, neglectful/rejecting, withholding support, terrorizing, exploitative).

	*Significant Factors*	*Beta*	*R (R* ^2^ *)*	*F*
Affective problems	Mother verbal abuse Neglectful/rejecting father Terrorizing father	.36*** .21* .18*	.691(.473)	90.328***
Anxiety problems	Neglectful/rejecting mother Neglectful/rejecting father	.35*** .24**	.572(.323)	72.380***
Attention deficit hyperactivity problems	Father verbal abuse Terrorizing mother	.37*** .26**	.603(.359)	84.866***
Oppositional defiant problems	Father verbal abuse Mother verbal abuse Terrorizing father	.20* .29*** .26**	.704(.490)	96.800***
Conduct problems	Neglectful/rejecting father Terrorizing father Mother verbal abuse	.29** .21* .19*	.706(.492)	73.391***
Obsessive compulsive problems	Mother verbal abuse Withholding support father	.25*** .14*	.327(.101)	17.73***
Post-traumatic stress problems	Mother verbal abuse Terrorizing father Withholding support father	.36*** .28*** .13*	.660(.430)	76.187***

B= Unstandardized Coefficient, SE= Std Error, β= Standardized regression Coefficients*.* R²= adjusted R^2^

*** p<.001,

** p<.01,

* p<.05,

## DISCUSSION

This study is an effort to identify the damaging effects of psychological abuse by parents on adolescents of Pakistan. This issue becomes even more important in developing countries like Pakistan where there is already lack of mental health services as well as recognition of damaging psychological abuse by parents. 

Findings of this are consistent with earlier researches which linked the PA to a variety of problems such as posttraumatic stress disorder, major depressive disorder, personality disorders, and low self-esteem,^21^ aggression, emotional unresponsiveness and neuroticism,^6^and anxiety, substance abuse**,** PTSD, and depression.^9^ In a retrospective study using a college sample Allen^22^ explored in regression analysis that terrorizing behaviors of parents, predicted anxiety and somatic complaints, ignoring parenting predicted depression and features of personality disorders, and degrading behaviors predicted features of personality disorders. Our research findings also supported earlier findings that parental PA is related with depression, anxiety, somatic complaints and other mental health problems. Findings are also consistent that verbal abuse is a leading risk factor for development of psychopathology.^23^

De Bellis^24^ argued that PTSD in children following abuse is the same condition as that diagnosed in adults following significant traumas, such as battlefield experiences or major accidents. American psychiatrists^25^ studied 156 children following severe abuse of parents, reported that 62 (40 per cent) met the diagnostic criteria for PTSD and 33% met the criteria even after two years, PTSD in turn also linked to internalizing disorders such as anxiety and depression and to externalizing disorders, such as ODD, ADHD and self-destructive behavior. Overall findings of the study are in agreement with earlier studies that parental abusive behaviors damage adolescents’ mental health. 

## Notes

***Limitations of the Current Study*** ***: ***Major limitations were reliance on retrospective responses of adolescents, small sample size, drawn from only urban area which may not be representative for whole population of children and adolescents. Prospective controlled studies are needed involving cross informants (parents & teachers), larger sample size including rural areas.

## Notes

***Conclusion: ***Present study revealed that abused adolescents were found vulnerable for mental health problems and this study is timely effort in the right direction to caution and guide parents, teachers, pediatricians and mental health professionals on the unrecognized psychological abuse and under reported mental health problems.

## Notes

***Acknowledgement: ***The authors appreciate the cooperation of the adolescents who participated in the study, their parents, school authorities and authors of scales, used in the study.

## Notes

***Note: ***This manuscript is based on PhD Dissertation “Perceived Parental Abuse, Parenting Styles, Associated Behavioral and Emotional Problems and Academic Achievement in Adolescents” by Syeda Fariha Iram Rizvi.
